# Supplementary material for: Language network lateralization is reflected throughout the macroscale functional organization of cortex
Source: Nat Commun. 2023 Jun 9;14:3405. doi: 10.1038/s41467-023-39131-y (PMC10256741; doi:10.1038/s41467-023-39131-y)
Supplement: Supplementary file 1 — Supplementary Information [file 41467_2023_39131_MOESM1_ESM.docx]

Supplementary Materials

Language network lateralization is reflected throughout the macroscale functional organization of cortex

**Authors:**

Loïc Labache^a,*^, Tian Ge^b, c, d^, B.T. Thomas Yeo^e, f, g, h, i^, Avram J. Holmes^a, j, k, l^

**Affiliations:**

^a^ Department of Psychology, Yale University, New Haven, US-CT 06520

^b^ Psychiatric and Neurodevelopmental Genetics Unit, Center for Genomic Medicine, Massachusetts General Hospital, Boston, US-MA 02114

^c^ Center for Precision Psychiatry, Department of Psychiatry, Massachusetts General Hospital, Boston, US-MA 02114

^d^ Stanley Center for Psychiatric Research, Broad Institute, Cambridge, US-MA 02142

^e^ Department of Electrical and Computer Engineering, Centre for Sleep and Cognition, National University of Singapore, Singapore, SG 119077

^f^ Department of Electrical and Computer Engineering, Centre for Translational Magnetic Resonance Research, National University of Singapore, Singapore, SG 119077

^g^ N.1 Institute for Health, National University of Singapore, Singapore, SG 119077

^h^ Martinos Center for Biomedical Imaging, Massachusetts General Hospital, Charlestown, US-MA 02129

^i^ National University of Singapore Graduate School for Integrative Sciences and Engineering, National University of Singapore, Singapore, SG 119077

^j^ Department of Psychiatry, Yale University, New Haven, US-CT 06520

^k^ Wu Tsai Institute, Yale University, New Haven, US-CT 06520

^l^ Department of Psychiatry, Brain Health Institute, Rutgers University, Piscataway, US-NJ, 08854

* Corresponding authors: Loïc Labache ([loic.labache@yale.edu](mailto:loic.labache@yale.edu)), and Avram J. Holmes ([avram.holmes@rutgers.edu](mailto:avram.holmes@rutgers.edu))

**Keywords:** Functional gradients, hemispheric specialization, brain lateralization, language network, cortical organization

# Demographic characterization according to language lateralization phenotype

Pearson's chi-squared test showed a significant effect of language lateralization phenotype on handedness (*χ*^2^=32.07, *p*<10^-4^). There were significantly more left-handers in the atypical group than in the strong typical (*χ*^2^=41.81, *p*<10^-4^), and than in the mild typical ones (*χ*^2^=23.83, *p*<10^-4^). No differences in proportion between mild and strong typical (*χ*^2^=3.50, *p*=0.061).

Pearson's chi-squared test showed a significant effect of language lateralization phenotype on sex (*χ*^2^=71.44, *p*<10^-4^). There were significantly more females in the mild atypical group than in the atypical (*χ*^2^= 9.71, *p*=0.002), and than in the strong typical group (*χ*^2^=71.17, *p*<10^-3^). No differences in proportion between atypical and strong typical (*χ*^2^=2.89, *p*=0.089).

Analysis of variance showed a significant effect of language lateralization phenotype on age (*p*<10^-3^). Mild typical individuals (*μ*=29.52 years, 95% confidence interval (CI)=±0.34 years) were older than atypical (*μ*=27.96 years, CI=±0.79 years, *p*=0.0012) and strong typical (*μ*=28.08 years, CI=±0.33 years, *p*<10^-4^). Atypical and strong typical individuals were the same age (*p*=0.96).

Analysis of variance showed a significant effect of language lateralization phenotype on educational level (*p*=0.033). Tukey’s HSD post-hoc test, which is conservative, reported no significant post-hocs tests. Strong typical individuals (*μ*=15.11 years, CI=±0.16 years) were not significantly different from mild typical (*μ*=14.86 years, CI=±0.17 years, *p*_corrected_=0.077, *p*_uncorrected_=0.03), or atypicals (*μ*=14.70 years, CI=±0.39 years, *p*_corrected_=0.12, *p*_uncorrected_=0.048). Atypical individuals were not significantly different from mild typical ones (*p*_corrected_=0.72, *p*_uncorrected_=0.44). That means that even if each pair of phenotypes were not significantly different from each other, there may exist a contrast (*i.e.* a combination or contrast of phenotypes) that is significant.

| Language lateralization phenotype | Demographics variables | |
| --- | --- | --- |
| Atypical | Sex | *n* = 41 female |
|  | Handedness | *n* = 26 left-handers |
|  | Average age (± 95% CI) | 27.96 ± 0.79 years |
|  | Average educational level (± 95% CI) | 14.70 ± 0.39 years |
| Mild typical | Sex | *n* = 294 female |
|  | Handedness | *n* = 48 left-handers |
|  | Average age (± 95% CI) | 29.52 ± 0.34 years |
|  | Average educational level (± 95% CI) | 15.11 ± 0.16 years |
| Strong typical | Sex | *n* = 192 female |
|  | Handedness | *n* = 36 left-handers |
|  | Average age | 28.08 ± 0.33 years |
|  | Average educational level (± 95% CI) | 14.86 ± 0.17 years |

**Supplementary Table 1.** HCP demographics according to language lateralization phenotype.

# Details of the ANCOVA results for the 5 features used to classify individuals

| Effect | *p*-value | Levels | Least Squares Mean/Regression slope | 95% Confidence Interval |
| --- | --- | --- | --- | --- |
| Age | 0.06 | - | - | - |
| Gender | 0.01 | female | 0.41 | ±0.11 |
|  |  | male | 0.58 | ±0.14 |
| Intracranial volume | 0.77 | - | - | - |
| Language lateralization phenotype | 2.85×10^-97^ | atypical | -0.96 | ±0.18 |
|  |  | mild-typical | 0.70 | ±0.12 |
|  |  | strong-typical | 1.74 | ±0.14 |
| Handedness | 3.76×10^-4^ | left-handers | 0.34 | ±0.15 |
|  |  | right-handers | 0.65 | ±0.08 |
| Language lateralization phenotype × Handedness | 0.14 | - | - | - |

**Supplementary Table 2.** ANCOVA’s results of the asymmetry during the language task at the network level. The displayed *p*-values correspond to that of the *F*-test.

| Effect | *p*-value | Levels | Least Squares Mean/Regression slope | 95% Confidence Interval |
| --- | --- | --- | --- | --- |
| Age | 0.15 | - | - | - |
| Gender | 0.21 | - | - | - |
|  |  |  |  |  |
| Intracranial volume | 0.63 | - | - | - |
| Language lateralization phenotype | 7.93×10^-94^ | atypical | -1.16 | ±0.26 |
|  |  | mild-typical | 1.17 | ±0.17 |
|  |  | strong-typical | 2.64 | ±0.19 |
| Handedness | 2.71×10^-4^ | left-handers | 0.66 | ±0.22 |
|  |  | right-handers | 1.11 | ±0.11 |
| Language lateralization phenotype × Handedness | 0.33 | - | - | - |

**Supplementary Table 3.** ANCOVA’s results of the asymmetry during the language task at the epicenters level (hubs). The displayed *p*-values correspond to that of the *F*-test.

| Effect | *p*-value | Levels | Least Squares Mean/Regression slope | 95% Confidence Interval |
| --- | --- | --- | --- | --- |
| Age | 0.82 | - | - | - |
| Gender | 0.76 | - | - | - |
|  |  |  |  |  |
| Intracranial volume | 0.99 | - | - | - |
| Language lateralization phenotype | 2.86×10^-19^ | atypical | 9 × 10^-3^ | ±0.18 |
|  |  | mild-typical | 0.85 | ±0.12 |
|  |  | strong-typical | 1.02 | ±0.13 |
| Handedness | 0.27 | - | - | - |
|  |  |  |  |  |
| Language lateralization phenotype × Handedness | 0.13 | - | - | - |

**Supplementary Table 4.** ANCOVA’s results of the strength asymmetry at rest. The displayed *p*-values correspond to that of the *F*-test.

| Effect | *p*-value | Levels | Least Squares Mean/Regression slope | 95% Confidence Interval |
| --- | --- | --- | --- | --- |
| Age | 9.94×10^-7^ | - | -0.10 | ±0.04 |
| Gender | 9.69×10^-7^ | female | 10.74 | ±0.31 |
|  |  | male | 11.67 | ±0.31 |
| Intracranial volume | 0.004 | - | 2×10^-6^ | ±1×10^-6^ |
| Language lateralization phenotype | 6.60×10^-27^ | atypical | 12.06 | ±0.53 |
|  |  | mild-typical | 9.4 | ±0.35 |
|  |  | strong-typical | 12.16 | ±0.39 |
| Handedness | 0.28 | - | - | - |
|  |  |  |  |  |
| Language lateralization phenotype × Handedness | 0.30 | - | - | - |

**Supplementary Table 5.** ANCOVA’s results of the strength sum at rest. The displayed *p*-values correspond to that of the *F*-test.

| Effect | *p*-value | Levels | Least Squares Mean/Regression slope | 95% Confidence Interval |
| --- | --- | --- | --- | --- |
| Age | 5.39×10^-8^ | - | -0.01 | ±2×10^-3^ |
| Gender | 0.01 | female | 0.56 | ±0.01 |
|  |  | male | 0.58 | ±0.02 |
| Intracranial volume | 0.04 | - | 5×10^-8^ | ±5×10^-8^ |
| Language lateralization phenotype | 1.33×10^-23^ | atypical | 0.61 | ±0.03 |
|  |  | mild-typical | 0.49 | ±0.02 |
|  |  | strong-typical | 0.61 | ±0.02 |
| Handedness | 0.75 | - | - | - |
|  |  |  |  |  |
| Language lateralization phenotype × Handedness | 0.13 | - | - | - |

**Supplementary Table 6.** ANCOVA’s results of the homotopic inter-hemispheric connectivity value at rest. The displayed *p*-values correspond to that of the *F*-test.

# Full description of ANCOVAs performed on gradient asymmetries

| Network | Gradient number | Typical  (*µ* ±CI) | Atypical  (*µ* ±CI) | *p-value* |
| --- | --- | --- | --- | --- |
| Default | 1 | **1.91 ±0.48** | **-1.62 ±1.00** | **5.72×10^-10^** |
|  | 2 | **-0.54 ±0.26** | **-1.58 ±0.53** | **5.97×10^-4^** |
|  | 3 | **-1.20 ±0.73** | **9.05 ±1.52** | **9.08×10^-31^** |
| Control | 1 | **-6.21 ±0.59** | **-9.21 ±1.21** | **1.48×10^-5^** |
|  | 2 | **-2.93 ±0.36** | **-4.26 ±0.74** | **0.002** |
|  | 3 | **-0.77 ±0.90** | **8.51 ±1.87** | **7.15×10^-18^** |
| Limbic | 1 | 2.88 ±0.75 | 1.76 ±1.55 | 0.20 |
|  | 2 | -0.63 ±0.48 | -1.28 ±0.99 | 0.25 |
|  | 3 | **-2.53 ±0.71** | **1.57 ±1.47** | **1.01×10^-6^** |
| Sal/VentrAttn | 1 | **1.11 ±0.51** | **-1.65 ±1.05** | **3.89×10^-6^** |
|  | 2 | **1.26 ±0.44** | **-0.17 ±0.92** | **0.006** |
|  | 3 | **-4.26 ±0.63** | **-1.25 ±1.31** | **5.19×10^-5^** |
| DorsAttn | 1 | **4.09 ±0.54** | **1.56 ±1.11** | **6.34×10^-5^** |
|  | 2 | 0.88 ±0.53 | 0.52 ±1.10 | 0.56 |
|  | 3 | -0.61 ±0.67 | 1.50 ±1.39 | 0.008 |
| Som/Motor | 1 | 0.01 ±0.24 | -0.02 ±0.50 | 0.91 |
|  | 2 | -2.52 ±0.41 | -1.77 ±0.85 | 0.12 |
|  | 3 | 0.34 ±0.34 | 0.84 ±0.71 | 0.22 |
| Visual | 1 | **1.60 ±0.26** | **0.67 ±0.53** | **0.002** |
|  | 2 | -0.24 ±0.29 | 0.29 ±0.61 | 0.12 |
|  | 3 | 0.53 ±0.28 | 1.12 ±0.58 | 0.07 |

**Supplementary Table 7.** Post-hoc results of the language lateralization main effect. Post-hoc analyses were conducted using a two-sided Student’s *t*-test. Bold cells are cells with a significant corrected effect (Bonferroni correction for network number, *p*<0.007). *µ* corresponds to the least-square mean value of the model. CI corresponds to the 95% Confidence Interval of *µ*.

|  | Default | Control | Limbic | Sal/VentAttn | DorsAttn | Som/Motor | Visual |
| --- | --- | --- | --- | --- | --- | --- | --- |
| R^2^_model_ (%) | 5.65 | 5.39 | 1.47 | 3.09 | 2.33 | 0.04 | 5.39 |
| *p-value*_model_ | 1.46×10^-10^ | 5.05×10^-10^ | 0.023 | 2.53×10^-5^ | 6.78×10^-4^ | 0.75 | 2.07×10^-3^ |
| Age | *p*=0.58 | *p*=0.68 | *p*=0.96 | *p*=0.22 | *p*=0.71 | *p*=0.11 | *p*=0.99 |
| ITV | *p*=0.61 | *p*=0.004  β=3 × 10^-6^ ±2 × 10^-6^ | *p*=0.49 | *p*=0.88 | *p*=0.83 | *p*=0.35 | *p*=0.79 |
| Gender | *p*=0.02 | *p*=0.26 | *p*=0.24 | *p*=0.16 | *p*=0.13 | *p*=0.70 | *p*=0.06 |
| MP | *p*=0.002  RH: 1.04 ±0.58  LH: -0.75 ±0.95 | *p*=0.19 | *p*=0.62 | *p*=0.36 | *p*=0.02 | *p*=0.91 | *p*=0.02 |
| MP x LLP | *p*=0.006  LH_atypical_: -3.3 ±1.65  RH_atypical_: 0.05 ±1.112  LH_typical_: 1.79 ±0.92  RH_typical_: 2.02 ±0.29 | *p*=0.78 | *p*=0.08 | *p*=0.61 | *p*=0.04 | *p*=0.76 | *p*=0.25 |

**Supplementary Table 8.** ANCOVA’s results of the first gradient asymmetries values. ITV corresponds to the Intracranial Volume (FreeSurfer-derived), MP to the handedness, LLP to Language Lateralization Phenotype, RH to Right-Handers, LH to Left-Handers. The least-square mean plus or minus the 95% confidence interval is reported when the main effect is significant. The displayed *p*-values correspond to that of the *F*-test.

|  | Default | Control | Limbic | Sal/VentAttn | DorsAttn | Som/Motor | Visual |
| --- | --- | --- | --- | --- | --- | --- | --- |
| R^2^_model_ (%) | 1.72 | 2.5 | 0.58 | 1.33 | 0.78 | 1.12 | 0.01 |
| *p-value*_model_ | 0.009 | 0.0003 | 0.45 | 0.04 | 0.26 | 0.083 | 0.19 |
| Age | *p*=0.99 | *p*=0.37 | *p*=0.42 | *p*=0.85 | *p*=0.63 | *p*=023 | *p*=0.27 |
| ITV | *p*=0.63 | *p*=0.01 | *p*=0.42 | *p*=0.79 | *p*=0.37 | *p*=0.31 | *p*=0.09 |
| Gender | *p*=0.86 | *p*=0.58 | *p*=0.41 | *p*=0.74 | *p*=0.60 | *p*=0.53 | *p*=0.47 |
| MP | *p*=0.01 | *p*=0.27 | *p*=0.97 | *p*=0.02 | *p*=0.02 | *p*=0.03 | *p*=0.26 |
| MP x LLP | *p*=0.02 | *p*=0.94 | *p*=0.49 | *p*=0.08 | *p*=0.50 | *p*=0.32 | *p*=0.07 |

**Supplementary Table 9.** ANCOVA’s results of the second gradient asymmetries values. ITV corresponds to the Intracranial Volume (FreeSurfer-derived), MP to the handedness, LLP to Language Lateralization Phenotype, RH to Right-Handers, LH to Left-Handers. The least-square mean plus or minus the 95% confidence interval is reported when the main effect is significant. The displayed *p*-values correspond to that of the *F*-test.

|  | Default | Control | Limbic | Sal/VentAttn | DorsAttn | Som/Motor | Visual |
| --- | --- | --- | --- | --- | --- | --- | --- |
| R^2^_model_ (%) | 17.23 | 12.94 | 3.60 | 5.86 | 1.76 | 1.70 | 1.65 |
| *p-value*_model_ | 1.12×10^-37^ | 4.13×10^-27^ | 2.49×10^-6^ | 5.18×10^-11^ | 0.007 | 0.01 | 0.01 |
| Age | *p*=0.17 | *p*=0.11 | *p*=0.64 | *p*=0.18 | *p*=0.46 | *p*=0.03 | *p*=0.01 |
| ITV | *p*=0.58 | *p*=0.19 | *p*=0.63 | *p*=0.08 | *p*=0.23 | *p*=0.32 | *p*=0.48 |
| Gender | *p*=0.02 | *p*=0.003  ♀: 4.85 ±1.23  ♂: 2.89 ±1.22 | *p*=0.29 | *p*=0.05 | *p*=0.32 | *p*=0.35 | *p*=0.91 |
| MP | *8.08×10^-7^*  RH: 1.79 ±0.89  LH: 6.06 ±1.44 | *p*=0.005  RH: 2.37 ±1.09  LH: 5.37 ±1.77 | *p*=0.10 | *p*=0.06 | *p*=0.08 | *p*=0.47 | *p*=0.07 |
| MP x LLP | *p*=0.011 | *p*=0.59 | *p*=0.46 | *p*=0.54 | *p*=0.04 | *p*=0.29 | *p*=0.59 |

**Supplementary Table 10.** ANCOVA’s results of the third gradient asymmetries values. ITV corresponds to the Intracranial Volume (FreeSurfer-derived), MP to the handedness, LLP to Language Lateralization Phenotype, RH to Right-Handers, LH to Left-Handers. The least-square mean plus or minus the 95% confidence interval is reported when the main effect is significant. The displayed *p*-values correspond to that of the *F*-test.

**
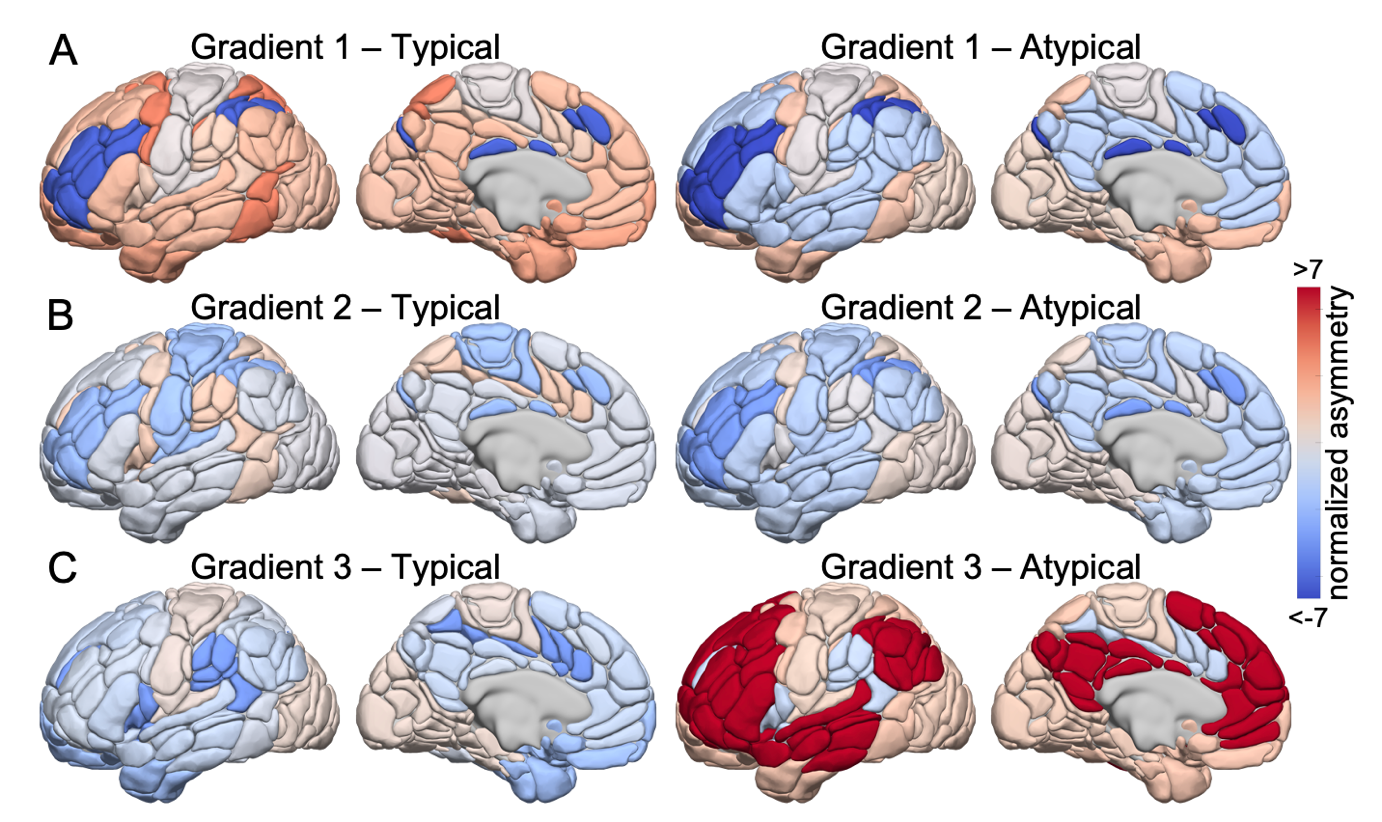
**

**Supplementary Figure 1.** Average gradient asymmetries values of functional connectivity across the cortical sheet (left *minus* right normalized gradient values). Asymmetries for typical individual (left hemisphere dominant for language) on the left, atypical individuals (right dominant) on the right. (**A**) Asymmetries values for the principal gradient of connectivity that transitions from the unimodal to the association cortex. (**B**) Asymmetries values for the second gradient that primarily differentiates the somato/motor and auditory cortex from the visual system. (**C**) Asymmetries values for the third gradient that reflects a network architecture contrasting frontoparietal from default and somato/motor systems. Warmer colors (solid red) indicate leftward asymmetries, colder colors (solid blue) reflects rightward asymmetries.

# Heritability of gradient asymmetry at the networks level

|  | Network | Heritability (*h*^2^) | Standard Error | *p-value* |
| --- | --- | --- | --- | --- |
| Gradient 1 | **Default *** | **0.22** | **0.06** | **7.17×10^-5^** |
|  | **Control *** | **0.19** | **0.06** | **1.11×10^-3^** |
|  | Limbic | 0.09 | 0.07 | 0.089 |
|  | **Sal/VentAttn *** | **0.25** | **0.06** | **1.17×10^-5^** |
|  | **DorsAttn *** | **0.2** | **0.06** | **5.85×10^-4^** |
|  | Som/Motor | 0.08 | 0.05 | 0.05 |
|  | Visual | 0.08 | 0.06 | 0.07 |
| Gradient 2 | **Default *** | **0.16** | **0.06** | **0.003** |
|  | **Control *** | **0.14** | **0.06** | **0.01** |
|  | Limbic | 0.03 | 0.05 | 0.28 |
|  | Sal/VentAttn | 0.08 | 0.06 | 0.08 |
|  | DorsAttn | 0.02 | 0.06 | 0.35 |
|  | Som/Motor | 0.06 | 0.06 | 0.14 |
|  | Visual | 0.02 | 0.05 | 0.32 |
| Gradient 3 | **Default *** | **0.28** | **0.06** | **2.10×10^-6^** |
|  | **Control *** | **0.28** | **0.06** | **1.10×10^-6^** |
|  | **Limbic *** | **0.16** | **0.06** | **0.003** |
|  | **Sal/VentAttn *** | **0.22** | **0.06** | **1.18×10^-4^** |
|  | **DorsAttn *** | **0.18** | **0.06** | **9.66×10^-4^** |
|  | Som/Motor | 0.00 | 0.00 | 0.50 |
|  | Visual | 0.09 | 0.06 | 0.06 |

**Supplementary t****able 11.** Heritability of individualized gradient values are greater in heteromodal (*h*^2^: *µ*=18.5%, SD=7.7%) relative to unimodal (*h*^2^: *µ*=5.5%, SD=3.8%) networks (*p*=0.001). Heritability of individual gradient value was estimated across 7 canonical functional networks using SOLAR[^70^](https://paperpile.com/c/RGtXhE/FyUOj). Raws in bold are those remaining significant effects after Bonferroni correction for network number (*p*≤0.007).

# Experimental workflow

**
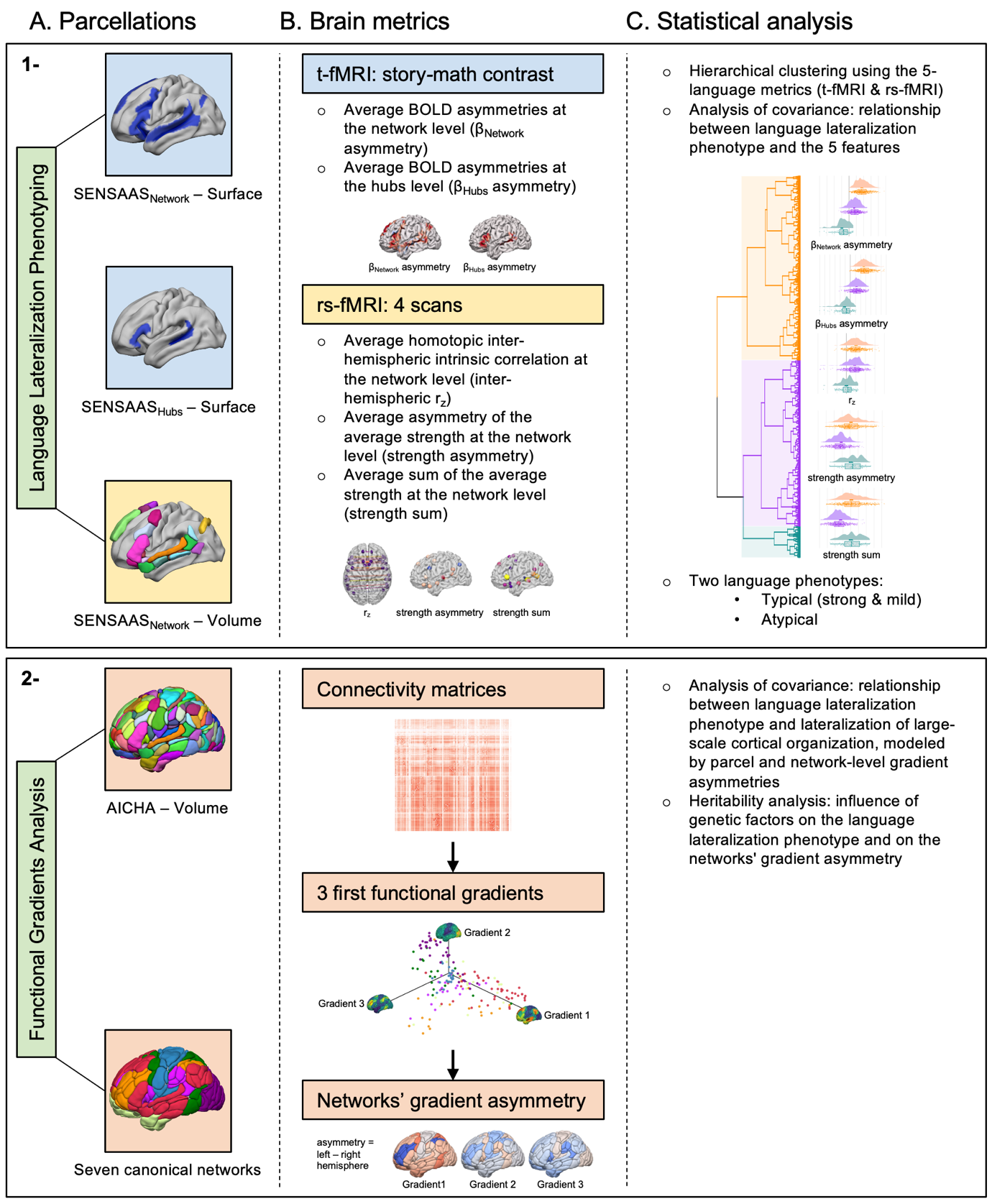
**

**Supplementary Figure 2.** Experimental workflow. (**A**) Different parcellations used. Box 1 (top to bottom): surface mask of the core high-order language atlas (SENSAAS, see Method – Language Atlas Statistics section for a full description), hubs of SENSAAS, SENSAAS in volume space. Box 2 (top to bottom): AICHA atlas, 7 canonical resting-state networks (see Method – Connectivity Embedding section for a full description). (**B**) Brain metrics used to characterize the participants (HCP, *n*=995). The 5 functional metrics of box 1 are fully described in the Method – Language Atlas Statistics section. The gradient embedding metrics presented in box 2 are fully described in the Method – Connectivity Embedding section. (**C**) Statistical analysis and their goals. Box 1: identification of the different language phenotypes is fully described in the Method – Statistical Analyses: Language Lateralization Identification section. Box 2: the analysis of covariance to analyze the impact of language lateralization on the gradient embedding is fully described in the Method – Statistical Analyses: Language Lateralization impact on gradient asymmetry section, the heritability analysis is fully described in the Method – Statistical Analyses: Heritability of Gradient Asymmetry and Language Lateralization Phenotype section. Only the left hemisphere of each parcellation and metrics is shown for illustration purposes.

# Replication of results using a different Edinburgh score

HCP’s Edinburgh Inventory (EHI) score is based on Schachter and colleagues[^1^](https://paperpile.com/c/RGtXhE/RgpfS), and includes a “footedness” item (see Supplementary Fig. [3A](#lsg5mdadvqxi)). Following recent recommendations from Raaf & Westerhausen[^2^](https://paperpile.com/c/RGtXhE/qHUYn), we computed an unbiased EHI score based. This corrected EHI is computed the same way, excluding the “footedness” item, and doubling the weight of the “writing” item (see Supplementary Fig. [3B](#lsg5mdadvqxi)). Both EHIs are highly and significantly correlated (r=0.99, p<10^-4^, Supplementary Fig. [3C](#lsg5mdadvqxi)), and there were no significant differences between them (Student's t-test: *μ*_difference_=-1.21, *t*=-0.60, *p*=0.55): highlighting the consistency of both metrics.

Using the same threshold as in the main analysis: an upper limit of 30 defines left-handedness. The unbiased EHI score leads to the following distribution: 110 left-handers and 885 right-handers. 3 individuals (2 mild typical and 1 strong typical) classified as right-handers with the HCP’s EHI score are now left-handers; likewise, 3 previously right-handers (2 mild typical and 1 strong typical) are now left-handers using the unbiased EHI score. Finally, the distribution of handedness among the groups does not change; 26 left-handers among 82 individuals in the atypical group, 48 left-handers among 433 in the mild typical group, and 36 left-handers among 480 individuals in the strong typical group.

We reanalyze the data with the unbiased EHI as a continuous covariate. The first re-analysis concerning the five features used for participants’ classification showed similar results (see Supplementary Table [12](#6ui0d8o2527)) to the one using the Handedness variable provided by the HCP.

Second, the new analysis of the main effect of language lateralization on gradient asymmetry (see Supplementary Table [13](#ijfeysmu6eou)) showed a broadly consistent pattern with minor differences concerning the networks with a significant effect after the Bonferroni correction. The asymmetry of the first gradient of the dorsal attentional network was no longer significant, and the same is true for the asymmetries of the second gradient of the default and salience/ventral attention networks. Critically, these results are concordant with our original analysis, highlighting the impact of language lateralization on the macroscale functional organization of the associative cortex.

Finally, the new analyses using the unbiased EHI as a continuous variable confirmed that the lateralization of the language network (*h*^2^=12.2%, SE=7%, *p*=0.028), and the hemispheric asymmetries in gradients organization are heritable (see Supplementary Table [14](#kix.kfq3fxlgm32x)). Furthermore, heritability at the network level remained consistent: heritability was more significant within heteromodal association cortices than within unimodal networks.


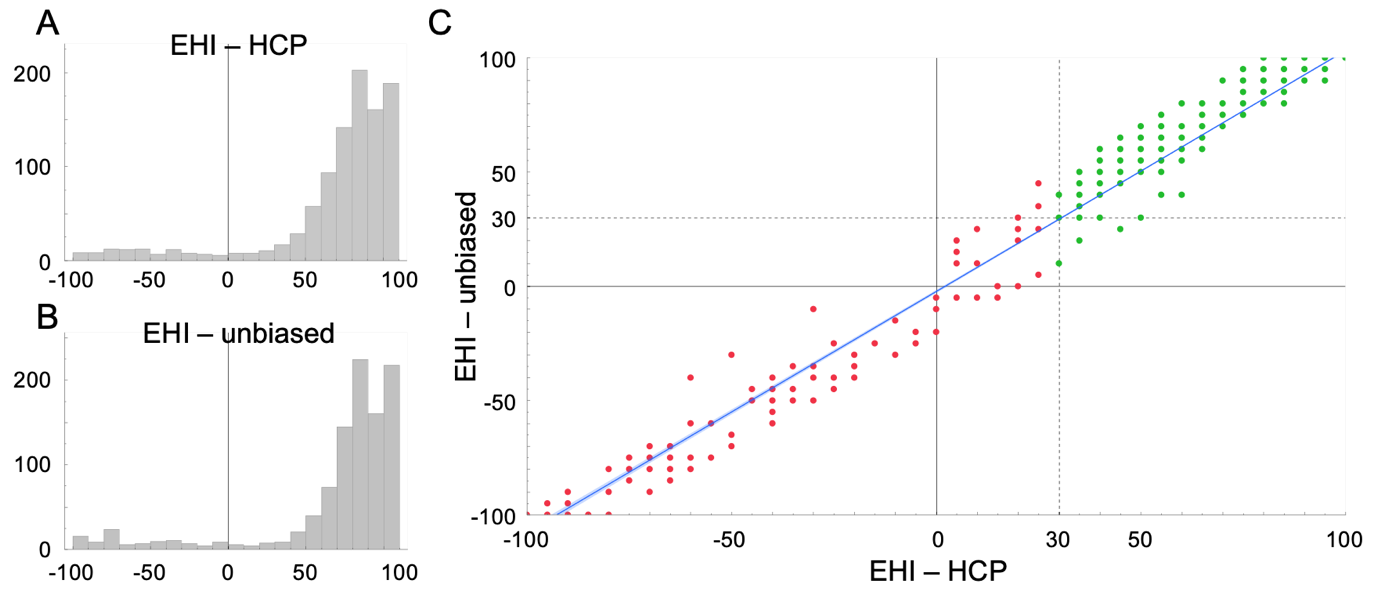


**Supplementary Figure 3.** Hand preference assessment in the HCP. (**A**) Edinburgh Inventory (EHI) scores distribution as provided by the HCP. (**B**) Unbiased Edinburgh Inventory score distribution. (**C**) Correlation between provided EHI score and unbiased EHI score. The EHI scores correlate significantly (*r*=0.99, *p*=2.37×10^-65^). Green dots are right-hander participants as defined based on the provided EHI score; red ones are left-hander. The blue line represents the linear regression line, and the blue light interval around the regression line represents the 95% confidence interval of the regression line. Vertical dashed line represents the left-handed definition threshold (<30, right-handed otherwise) for the HCP's EHI score. The horizontal dashed line represents the left-handed definition threshold (<30, right-handed otherwise) for the unbiased EHI score. The solid vertical and horizontal lines represent the handedness definition with a threshold of 0 (<0 defines left-handedness, right-hander otherwise).

| Variable | Language lateralization phenotype (LLP) effect (mean ± CI_95%_) | Post-hoc (p-value) | p-value |
| --- | --- | --- | --- |
| β_Network_ asymmetry | *μ_strong typical_* = 1.79 ± 0.07  *μ_mild typical_* = 0.78 ± 0.08  *μ_atypical_* = -0.74 ± 0.19 | *μ_strong typical_* > *μ_mild typical_*: 4.00×10^-11^  *μ_mild typical_* > *μ_atypical_*: 4.00×10^-11^  *μ_strong typical_* > *μ_atypical_*: 4.00×10^-11^ | p_model_: 7.40×10^-148^  p_LLP_: 8.90×10^-126^ |
| β_Hubs_ asymmetry | *μ_strong typical_* = 2.79 ± 0.10  *μ_mild typical_* = 1.27 ± 0.11  *μ_atypical_* = -0.89 ± 0.27 | *μ_strong typical_* > *μ_mild typical_*: 4.00×10^-11^  *μ_mild typical_* > *μ_atypical_*: 4.00×10^-11^  *μ_strong typical_* > *μ_atypical_*: 4.00×10^-11^ | p_model_: 7.70×10^-148^  p_LLP_: 1.30×10^-128^ |
| Inter-hemispheric r_Z_ | *μ_strong typical_* = 0.60 ± 0.01  *μ_mild typical_* = 0.49 ± 0.01  *μ_atypical_* = 0.63 ± 0.03 | *μ_strong typical_* > *μ_mild typical_*: 4.00×10^-11^  *μ_mild typical_ <* *μ_atypical_*: 4.00×10^-11^  *μ_strong typical_ =* *μ_atypical_*: 0.084 | p_model_: 4.26×10^-79^  p_LLP_: 7.99×10^-53^ |
| Strength asymmetry | *μ_strong typical_* = 1.00 ± 0.07  *μ_mild typical_* = 0.83 ± 0.07  *μ_atypical_* = 0.14 ± 0.14 | *μ_strong typical_* > *μ_mild typical_*: 3.60×10^-3^  *μ_mild typical_* > *μ_atypical_*: 6.00×10^-11^  *μ_strong typical_* > *μ_atypical_*: 4.00×10^-11^ | p_model_: 2.12×10^-19^  p_LLP_: 7.34×10^-17^ |
| Strength sum | *μ_strong typical_* = 12.17 ± 0.21  *μ_mild typical_* = 9.37 ± 0.22  *μ_atypical_* = 12.37 ± 0.54 | *μ_strong typical_* > *μ_mild typical_*: 4.00×10^-11^  *μ_mild typical_* < *μ_atypical_*: 4.00×10^-11^  *μ_strong typical_* = *μ_atypical_*: 0.77 | p_model_: 1.50×10^-103^  p_LLP_: 5.96×10^-63^ |

**Supplementary table 12.** ANCOVA results of the 5 functional features to classify participants. Significance of the Language Lateralization Phenotype main effect. The displayed *p*-values (in the column of the same name) correspond to that of the *F*-test. The *p*-values in the Post-hoc column correspond to two-sided Student’s *t*-test *p*-values.

|  | Network | Language lateralization phenotype (LLP) effect (mean ± CI_95%_) | Post-hoc: *t* (*p*-value) | *p*-value |
| --- | --- | --- | --- | --- |
| G1 | **Default *** | ***μ_typical_* = 1.99 ± 0.28**  ***μ_atypical_* = -0.30 ± 0.30** | ***μ_typical_* > *_atypical_*: -4.24 (2.43×10^-5^)** | **p_model_: 1.63×10^-10^**  **p_LLP_: 2.43×10^-5^** |
|  | **Control *** | ***μ_typical_* = -5.78 ± 0.34**  ***μ_atypical_* = -8.79 ± 1.25** | ***μ_typical_ >* *_atypical_*: -4.56 (5.67×10^-6^)** | **p_model_: 5.02×10^-10^**  **p_LLP_: 5.67×10^-6^** |
|  | Limbic | *μ_typical_* = 3.31 ± 0.43  *μ_atypical_* = 1.19 ± 1.19 | *μ_typical_* > *_atypical_*: -2.51 (0.0121) | p_model_: 0.0424  p_LLP_: 0.0121 |
|  | **Sal/VentAttn *** | ***μ_typical_* = 1.20 ± 0.29**  ***μ_atypical_* = -1.28 ± 1.08** | ***μ_typical_* > *_atypical_*: -4.35 (1.52×10^-^)** | **p_model_: 1.32×10^-5^**  **p_LLP_: 1.52×10^-5^** |
|  | DorsAttn | *μ_typical_* = 4.17 ± 0.31  *μ_atypical_* = 2.71 ± 1.14 | *μ_typical_* > *_atypical_*: -2.42 (0.0157) | p_model_: 0.0004  p_LLP_: 0.0157 |
|  | Som/Motor | *μ_typical_* = 0.03 ± 0.03  *μ_atypical_* = -0.05 ± 0.05 | *μ_typical_* > *_atypical_*: -0.31 (0.7562) | p_model_: 0.7619  p_LLP_: 0.7562 |
|  | Visual | *μ_typical_* = 1.74 ± 0.15  *μ_atypical_* = 1.17 ± 0.55 | *μ_typical_* > *_atypical_*: -1.97 (0.0491) | p_model_: 0.0010  p_LLP_: 0.0491 |
| G2 | Default | *μ_typical_* = -0.51 ± 0.15  *μ_atypical_* = -0.95 ± 0.55 | *μ_typical_* > *_atypical_*: -1.52 (0.1278) | p_model_: 0.0079  p_LLP_: 0.1278 |
|  | **Control *** | ***μ_typical_* = -2.76 ± 0.21**  ***μ_atypical_* = -3.97 ± 0.76** | ***μ_typical_* > *_atypical_*: -3.01 (0.0026)** | **p_model_: 0.0002**  **p_LLP_: 0.0026** |
|  | Limbic | *μ_typical_* = -0.47 ± 0.28  *μ_atypical_* = -1.44 ± 1.02 | *μ_typical_* > *_atypical_*: -1.80 (0.0717) | p_model_: 0.4801  p_LLP_: 0.0717 |
|  | Sal/VentAttn | *μ_typical_* = 1.39 ± 0.26  *μ_atypical_* = 0.67 ± 0.67 | *μ_typical_* > *_atypical_*: -1.44 (0.1500) | p_model_: 0.0292  p_LLP_: 0.1500 |
|  | DorsAttn | *μ_typical_* = 1.27 ± 0.31  *μ_atypical_* = 1.37 ± 1.13 | *μ_typical_* < *_atypical_*: 0.16 (0.8737) | p_model_: 0.1096  p_LLP_: 0.8737 |
|  | Som/Motor | *μ_typical_* = -2.30 ± 0.24  *μ_atypical_* = -1.15 ± 0.87 | *μ_typical_* < *_atypical_*: 2.50 (0.0124) | p_model_: 0.0291  p_LLP_: 0.0124 |
|  | Visual | *μ_typical_* = -0.14 ± 0.14  *μ_atypical_* = -0.11 ± 0.11 | *μ_typical_* < *_atypical_*: 0.10 (0.9213) | p_model_: 0.1747  p_LLP_: 0.9213 |
| G3 | **Default *** | ***μ_typical_* = -2.02 ± 0.42**  ***μ_atypical_* = 6.44 ± 1.56** | ***μ_typical_* < *_atypical_*: 10.27 (1.37×10^-23^)** | **p_model_: 1.10×10^-37^**  **p_LLP_: 1.37×10^-23^** |
|  | **Control *** | ***μ_typical_* = -1.72 ± 0.52**  ***μ_atypical_* = 7.01 ± 1.92** | ***μ_typical_* < *_atypical_*: 8.63 (2.51×10^-17^)** | **p_model_: 1.09×10^-27^**  **p_LLP_: 2.51×10^-17^** |
|  | **Limbic *** | ***μ_typical_* = -2.83 ± 0.41**  ***μ_atypical_* = 0.74 ± 0.74** | ***μ_typical_* < *_atypical_*: 4.47 (8.54×10^-6^)** | **p_model_: 3.20×10^-6^**  **p_LLP_: 8.54×10^-6^** |
|  | **Sal/VentAttn *** | ***μ_typical_* = -5.00 ± 0.37**  ***μ_atypical_* = -1.65 ± 1.35** | ***μ_typical_* < *_atypical_*: 4.71 (2.83×10^-6^)** | **p_model_: 3.64×10^-10^**  **p_LLP_: 2.83×10^-6^** |
|  | DorsAttn | *μ_typical_* = -1.08 ± 0.39  *μ_atypical_* = 0.85 ± 0.85 | *μ_typical_* < *_atypical_*: 2.55 (0.0108) | p_model_: 0.0116  p_LLP_: 0.0108 |
|  | Som/Motor | *μ_typical_* = 0.29 ± 0.20  *μ_atypical_* = 1.14 ± 0.73 | *μ_typical_* < *_atypical_*: 2.19 (0.0288) | p_model_: 0.0053  p_LLP_: 0.0288 |
|  | Visual | *μ_typical_* = 0.37 ± 0.16  *μ_atypical_* = 0.82 ± 0.60 | *μ_typical_* < *_atypical_*: 1.45 (0.1487) | p_model_: 0.0165  p_LLP_: 0.1487 |

**Supplementary table 13.** ANCOVA’s results of the three gradient asymmetries values. Significance of the Language Lateralization Phenotype main effect. Colored lines display significant uncorrected effects (*p*≤0.05). Bold lines with a star are significant after Bonferroni correction for the number of networks (*p*≤0.007). The displayed *p*-values (in the column of the same name) correspond to that of the *F*-test. The *p*-values in the Post-hoc column correspond to two-sided Student’s *t*-test *p*-values.

|  | Network | Heritability (*h*^2^) | Standard Error | *p-*value |
| --- | --- | --- | --- | --- |
| G1 | **Default *** | **0.21** | **0.06** | **0.0001** |
|  | **Control *** | **0.19** | **0.06** | **0.0014** |
|  | Limbic | 0.09 | 0.07 | 0.0884 |
|  | **Sal/VentAttn *** | **0.25** | **0.06** | **1.22×10^-5^** |
|  | **DorsAttn *** | **0.19** | **0.06** | **0.0008** |
|  | Som/Motor | 0.08 | 0.05 | 0.0537 |
|  | Visual | 0.08 | 0.06 | 0.0810 |
| G2 | **Default *** | **0.16** | **0.06** | **0.0034** |
|  | Control | 0.13 | 0.06 | 0.0092 |
|  | Limbic | 0.03 | 0.05 | 0.2784 |
|  | Sal/VentAttn | 0.07 | 0.06 | 0.1139 |
|  | DorsAttn | 0.02 | 0.06 | 0.3392 |
|  | Som/Motor | 0.06 | 0.06 | 0.1692 |
|  | Visual | 0.02 | 0.05 | 0.3178 |
| G3 | **Default *** | **0.29** | **0.06** | **9.00×10^-7^** |
|  | **Control *** | **0.28** | **0.06** | **7.00×10^-7^** |
|  | **Limbic *** | **0.16** | **0.06** | **0.0023** |
|  | **Sal/VentAttn *** | **0.22** | **0.06** | **9.61×10^-5^** |
|  | **DorsAttn *** | **0.19** | **0.06** | **0.0008** |
|  | Som/Motor | 0.00 | 0.00 | 0.5000 |
|  | Visual | 0.10 | 0.06 | 0.0501 |

**Supplementary table 14.** Heritability of individualized gradient values. Colored lines reflect networks with significant heritability of gradient laterality (*p*≤0.05 uncorrected). Heritability of individual gradient value was estimated across 7 canonical functional networks using SOLAR[^70^](https://paperpile.com/c/RGtXhE/FyUOj). Lines with a star remain significant after Bonferroni correction for the number of networks (*p*≤0.007).

References

1. [Schachter, S. C., Ransil, B. J. & Geschwind, N. Associations of handedness with hair color and learning disabilities. *Neuropsychologia* **25**, 269–276 (1987).](http://paperpile.com/b/RGtXhE/RgpfS)

2. [Raaf, N. & Westerhausen, R. Hand preference and the corpus callosum: Is there really no association? *Neuroimage Rep.* **3**, 100160 (2023).](http://paperpile.com/b/RGtXhE/qHUYn)
