# Supplementary material for: Language network lateralization is reflected throughout the macroscale functional organization of cortex
Source: Nat Commun. 2023 Jun 9;14:3405. doi: 10.1038/s41467-023-39131-y (PMC10256741; doi:10.1038/s41467-023-39131-y)
Supplement: Supplementary file 3 — Reporting Summary [file 41467_2023_39131_MOESM3_ESM.pdf]

nature portfolio

Corresponding author(s): Loic Labache

Last updated by author(s): Apr 26, 2023

Reporting Summary

Nature Portfolio wishes to improve the reproducibility of the work that we publish. This form provides structure for consistency and transparency in reporting. For further information on Nature Portfolio policies, see our Editorial Policies and the Editorial Policy Checklist.

Statistics

For all statistical analyses, confirm that the following items are present in the figure legend, table legend, main text, or Methods section.

n/a	Confirmed	
☐	☒	The exact sample size (*n*) for each experimental group/condition, given as a discrete number and unit of measurement
☐	☒	A statement on whether measurements were taken from distinct samples or whether the same sample was measured repeatedly
☐	☒	The statistical test(s) used AND whether they are one- or two-sided<br>*Only common tests should be described solely by name; describe more complex techniques in the Methods section.*
☐	☒	A description of all covariates tested
☐	☒	A description of any assumptions or corrections, such as tests of normality and adjustment for multiple comparisons
☐	☒	A full description of the statistical parameters including central tendency (e.g. means) or other basic estimates (e.g. regression coefficient) AND variation (e.g. standard deviation) or associated estimates of uncertainty (e.g. confidence intervals)
☐	☒	For null hypothesis testing, the test statistic (e.g. *F*, *t*, *r*) with confidence intervals, effect sizes, degrees of freedom and *P* value noted<br>*Give P values as exact values whenever suitable.*
☒	☐	For Bayesian analysis, information on the choice of priors and Markov chain Monte Carlo settings
☒	☐	For hierarchical and complex designs, identification of the appropriate level for tests and full reporting of outcomes
☐	☒	Estimates of effect sizes (e.g. Cohen's *d*, Pearson's *r*), indicating how they were calculated

Our web collection on statistics for biologists contains articles on many of the points above.

Software and code

Policy information about availability of computer code

Data collection	No software used for data collection.
Data analysis	We utilized widely used R libraries and customized R code for this study. The R libraries includes: R (version 4.1.0), dplyr (version 1.0.10), oro.nifti (version 0.11.4), RNifti (version 1.3.1), neurohcp (version 0.9.0), . The code utilized have be made publicly available on Github at link: https://github.com/loiclabache/Labache_2022_AO. We also used Python (version 3.8.10) alongside the BrainSpace library (version 0.1.3). The Surf Ice software was used to realize brain visualization (version: 6 october 2021, https://www.nitrc.org/projects/surfice/).

For manuscripts utilizing custom algorithms or software that are central to the research but not yet described in published literature, software must be made available to editors and reviewers. We strongly encourage code deposition in a community repository (e.g. GitHub). See the Nature Portfolio guidelines for submitting code & software for further information.

Data

Policy information about availability of data

All manuscripts must include a data availability statement. This statement should provide the following information, where applicable:

- Accession codes, unique identifiers, or web links for publicly available datasets
- A description of any restrictions on data availability
- For clinical datasets or third party data, please ensure that the statement adheres to our policy

This study used publicly available data from the HCP (https://www.humanconnectome.org/). Data can be accessed via data use agreements. The language atlas is available here: https://github.com/loiclabache/SENSAAS_brainAtlas.

Human research participants

Policy information about studies involving human research participants and Sex and Gender in Research.

Reporting on sex and gender	In the HCP, the information of gender is self-reported by each participant. In our study, the sample is balanced in gender with 48% of women (447 individuals). In the HCP dataset, gender information and related consent have been collected and obtained from all subjects (477 women, 518 men). Gender has been used as covariate in the different analyses as reported in the method section. Gender-based analysis has not been directly performed in this study. Gender analysis haven't been performed here since gender is not associated with the occurrence of critical changes in language lateralization, even though previous reports have noted a reduced language lateralization in women.
Population characteristics	This study utilized data from the WU-Minn HCP Consortium S1200 Release. HCP S1200 release comprised 1206 healthy young adults (age 22-35, 657 female). A sub-sample of 995 participants has been used in this study (mean age=28.7, sd=3.71 years, 477 women).
Recruitment	For the HCP S1200 release, 1206 healthy young adult (age 22-35) participants were recruited from families with twins and non-twin siblings in Human Connectome Project (HCP). Authors are not involved in the recruitment of the datasets. More information can be found at https://www.humanconnectome.org/study/hcp-young-adult/project-protocol/recruitment.
Ethics oversight	Although the data was not collected by us, the current study was approved by the Yale University IRB.

Note that full information on the approval of the study protocol must also be provided in the manuscript.

Field-specific reporting

Please select the one below that is the best fit for your research. If you are not sure, read the appropriate sections before making your selection.

[x] Life sciences [ ] Behavioural & social sciences [ ] Ecological, evolutionary & environmental sciences

For a reference copy of the document with all sections, see nature.com/documents/nr-reporting-summary-flat.pdf

Life sciences study design

All studies must disclose on these points even when the disclosure is negative.

Sample size	From the initial S1200 release of the HCP, healthy participants with fully completed 3T language and 3T resting-state fMRI protocols were selected, resulting in a total of 995 participants. No sample size calculation was performed. The current sample size included all participants with data that survived the criteria described below.
Data exclusions	Participants were included if they had 1) language task data and 2) resting-state functional MR images 3) had complete information for essential interests (age, gender, years of education, Edinburgh score for handedness).
Replication	According to the specificity of the actual study (language and resting-state fMRI required) no replication have been done regarding the language lateralization effect on the gradient asymmetries. It has to be noted that the results concerning the classification of individuals (typical or atypical) is a direct replication of a previous paper (see Labache et al, 2020, Elife, DOI: 10.7554/eLife.58722).
Randomization	We utilized the publicly available HCP dataset in our study, which was not randomized. Randomization is not pertinent to our current investigation, as the statistical method employed (hierarchical classification) does not necessitate randomization. The objective is to classify individuals in the dataset based on a similarity metric, and no state-of-the-art hierarchical classification methods require randomization. Furthermore, we conducted ANCOVA analyses to determine the impact of specific phenotypes on various brain metrics, which also do not require randomization. In this study, we did not use any statistical techniques such as prediction, that call for randomization.
Blinding	Group analyses include all participants surviving the inclusion criteria. Furthermore, blinding is not relevant to this study as no data collection was involved.

Reporting for specific materials, systems and methods

We require information from authors about some types of materials, experimental systems and methods used in many studies. Here, indicate whether each material, system or method listed is relevant to your study. If you are not sure if a list item applies to your research, read the appropriate section before selecting a response.

Materials & experimental systems

n/a	Involved in the study
☒	☐ Antibodies
☒	☐ Eukaryotic cell lines
☒	☐ Palaeontology and archaeology
☒	☐ Animals and other organisms
☒	☐ Clinical data
☒	☐ Dual use research of concern

Methods

n/a	Involved in the study
☒	☐ ChIP-seq
☒	☐ Flow cytometry
☐	☒ MRI-based neuroimaging

Magnetic resonance imaging

Experimental design

Design type: Resting-state functional MRI and task functional MRI. One task was included: anguage processing tasks, and all task fMRI in HCP are block design.

Design specifications: The experimental paradigms for task fMRI scanning were follow the HCP protocols.

Behavioral performance measures: Only the Edinburgh inventory has been use in this study to define manual prefrence (mean=66.5, sd=43.3, range=[-100; 100].

Acquisition

Imaging type(s): functional MRI (task and resting-state)

Field strength: Siemens 3T MRI scanner (Skyra system)

Sequence & imaging parameters: multiband EPI sequence. Repetition time (TR)=720ms, echo time (TE)=33.1ms, voxel dimension: 2mm isotropic. Details on fMRI can be found elsewhere: https://www.humanconnectome.org/study/hcp-young-adult/document/1200-subjects-data-release.

Area of acquisition: Whole brain scan was used.

Diffusion MRI: ☐ Used ☒ Not used

Preprocessing

Preprocessing software: HCP minimal preprocessing pipelines (S1200 release, march 2017)

Normalization: Details on preprocessing can be found in Glasser et al 2013, DOI: 10.1016/j.neuroimage.2013.04.127.

Normalization template: Details on preprocessing can be found in Glasser et al 2013, DOI: 10.1016/j.neuroimage.2013.04.127.

Noise and artifact removal: Details on preprocessing can be found in Glasser et al 2013, DOI: 10.1016/j.neuroimage.2013.04.127.

Volume censoring: Details on preprocessing can be found in Glasser et al 2013, DOI: 10.1016/j.neuroimage.2013.04.127.

Statistical modeling & inference

Model type and settings: See Barch et al 2013, DOI: 10.1016/j.neuroimage.2013.05.033.

Effect(s) tested: See Barch et al 2013, DOI: 10.1016/j.neuroimage.2013.05.033.

Specify type of analysis: ☐ Whole brain ☐ ROI-based ☒ Both

Anatomical location(s): We used the language atlas previously published in Labache et al, 2019, Brain Structure and Function, DOI: 10.1007/s00429-018-1810-2. Whole brain analysis has been performed using the AICHA atlas (see Joliot et al 2015, DOI: 10.1016/j.jneumeth.2015.07.013.

Statistic type for inference<br>(See Eklund et al. 2016)	Analysis of covariance, post-hoc analyses were conducted using Tukey's range test for multiple comparisons, or Student's t-test for binary ones.
Correction	Bonferroni was employed in this study.

Models & analysis

n/a	Involved in the study
☐	☒ Functional and/or effective connectivity
☐	☒ Graph analysis
☒	☐ Multivariate modeling or predictive analysis

Functional and/or effective connectivity	Functional connectivity was computed using the Pearson correlation. Fisher z-transformed when needed.
Graph analysis	Centrality degree (or strength) has been computed for each brain regions belonging to the language network (n=18) as the sum of the positive correlations existing between one region and all the 18 others.
